# Quality assurance for care of the dying: engaging with clinical services to facilitate a regional cross-sectional survey of bereaved relatives’ views

**DOI:** 10.1186/s12913-018-3558-z

**Published:** 2018-10-10

**Authors:** Catriona Mayland, Tamsin McGlinchey, Maureen Gambles, Helen Mulholland, John Ellershaw

**Affiliations:** 1 Department of Oncology and Metabolism, University of Sheffield, Broomcross Building, Whitham Road, Sheffield, S10 2SJ UK; 2 Palliative Care Institute, University of Liverpool, Cancer Research Centre, 200 London Road, Liverpool, L3 9TA UK; 3 Royal Liverpool & Broadgreen University NHS Hospitals Trust, Prescot Street, Liverpool, L7 8XP UK

**Keywords:** Quality of healthcare, Proxy, Bereavement, Healthcare surveys, Palliative care

## Abstract

**Background:**

Globally, having the ‘patient and /or family voice’ engaged when measuring quality of care for the dying is fundamentally important. This is particularly pertinent within the United Kingdom, where changes to national guidance about care provided to dying patients has heightened the importance of quality assurance and user-feedback. Our main aim was to engage with clinical services (hospice, hospital and community settings) within a specific English region and conduct a bereaved relatives’ cross-sectional survey about quality of care. Our secondary aim was to explore levers and barriers to project participation as perceived by organisational representatives.

**Methods:**

Each organisation identified a consecutive sample of next-of-kin to adult patients who died between 1st September and 30th November 2014. Those who had an unexpected death or were involved in a formal complaint were excluded. The ‘Care Of the Dying Evaluation’ (CODE™) questionnaire was posted out three months following the bereavement. One-to-one interviews were undertaken with a purposive sample of organisational representatives to explore experiences about project participation.

**Results:**

Of the 30 invited organisations, 18 were able to participate comprising: 7 hospitals, 7 hospices and 4 community settings. There were 1774 deaths which met the inclusion criteria but 460 (26%) were excluded due to inaccurate next-of-kin details. Subsequently, 1283 CODE™ questionnaires were sent out, with 354 completed (27% response rate). Overall, most participants perceived good quality of care. A notable minority reported poor care for symptom control and communication especially within the hospital. Nine interviews were conducted - levers to project participation included the ‘significance of user-feedback and the opportunity to use results in a meaningful way’; the main barrier was related to ‘concern about causing distress to bereaved relatives’.

**Conclusions:**

Overall, being able to engage with 18 (60%) organisations within the region and conduct the bereaved relatives’ survey showed success of this initiative and was supported by interview findings. The potential to be able to benchmark user-feedback against other organisations was thought to help focus on areas to develop services. This type of quality assurance project could form a template model and be replicated on a national and international level.

## Background

Calls to ensure the ‘patient and family voice’ is encapsulated into the measurement of quality of care for the dying is well-established [1]. Hence, on a global basis, bereaved relatives’ evaluations (both using surveys and telephone interviews) form a key part of the evaluation of end-of-life care, especially within North America, Japan, and parts of Europe [2–6]. To ensure the highest level of care provision, it is important to be able to robustly evaluate the quality of current care [7].

This evaluation is especially pertinent within the United Kingdom (UK), as within recent years, care for dying patients has featured heavily within public and professional forums. Additionally, significant changes have occurred to national guidance underpinning the way that care should be provided [8, 9]. The Liverpool Care Pathway for the Dying Patient (LCP) [10], an integrated care pathway aiming to improve quality of care in the last days of life, came under intense media scrutiny. The LCP was a nationally endorsed document and used internationally to support the provision of care when it was recognised that an individual may be in the last days of life. A subsequent review of care for the dying advised that the LCP should be phased out by July 2014 [8]. Forty-four key recommendations were provided within the report, including the need for more individualised care and improving skills and competencies for clinical staff caring for dying patients [8]. Following this, the ‘One chance to get it right’ report identified five ‘priorities for care’ and highlighted that clear, sensitive, and timely communication is fundamental to ensure good quality of care is provided to dying patients and their families [9]. A recommendation for ‘individualised end-of-life care plans’ was made and the recent publication of the NICE guidelines for End-of-life care helped provide a framework for best clinical practice [11].

The most recent National UK ‘End of Life Care Audit – Dying in Hospital’ based on data collected in 2015, demonstrated improvements compared with the previous audit in 2013: a higher proportion of patients were recognised to be dying in a timely manner and for 95% of these, there was a documented discussion about this recognition with those identified as important to the patient [12]. There was, however, a reduction in the rates of anticipatory prescribing for symptoms commonly seen in the last days of life. While this could be perceived as positive and in keeping with NICE guidelines about individualised prescribing, a regional survey of first year doctors reported one of their main needs was for formal guidance with symptom control. These doctors reported difficulties with remembering doses of anticipatory medication [13]. Importantly, the 2015 National audit also highlighted variability in the results between individual hospitals and the continued limited availability of 24/7 palliative care services [12].

In view of these major changes in end-of-life healthcare policy, a quality assurance project was undertaken within a specific English region to assess current care provided to dying patients and their families in three healthcare settings: hospital, hospice, and community. In order to focus on care in the last days of life and immediate post-bereavement period, we used the ‘Care Of the Dying Evaluation’ (CODE™) questionnaire [14] as our post-bereavement tool. CODE™ seeks perceptions about quality of patient care and level of family support and contains sections on symptom control; nursing and medical care; communication; provision of fluids; place of death; and emotional and spiritual support. It is a shortened version of the original questionnaire, ‘Evaluating Care and Health Outcomes – for the Dying (ECHO-D), which was developed, validated and used within a hospice and hospital setting [15–17]. CODE™ has also been assessed for validity and reliability [14], has been used nationally within the National Care of the Dying Audit – Hospitals [18], and is currently the focus of an international project involving seven European and Latin American counties [19].

Undertaking robust research for those who are dying is challenging due to the sensitive and emotive area of enquiry. Ethical, moral and practical challenges exist, influencing recruitment, retention, difficulty in identifying suitable outcomes measures and the level of investment in research [20–24]. With this in mind and key for the context of this quality assurance project, it is important to identify a distinction between measurement for judgement and measurement for improvement. It has been argued that in order to facilitate the process of service improvement “we need just enough information to take a next step in learning” [25]. Benchmarking methodology offers a way in which to generate ‘just enough information’, through facilitating assessment, comparison and reflection of ‘relevant’ information on care delivery, to identify both gaps in performance and examples of best practice [26, 27]. This methodology promotes a collaborative rather than competitive approach to assessment focussing on sharing information, which is integral to continually improving the quality of care [27]. This ethos underpins the methodology of this reported quality assurance project.

### Aims

Within a specific English region, the primary aim was to engage with clinical services across hospital, hospice and community settings to explore the current quality of care provided to dying patients and their families, from the perspective of bereaved relatives. In order to achieve this aim, we undertook a cross-sectional regional survey of bereaved relatives’ using the CODE™ questionnaire. The project was known as the Regional CODE™ survey and the conduct and results from this survey represents the main focus of this manuscript.

As a secondary aim, we wanted to explore views on project participation as perceived by representatives from individual organisations. To achieve this aim, one-to-one evaluation interviews with organisational representatives were undertaken. Within the context of this manuscript, we will focus on summarising key feedback about the levers and barriers to project participation.

## Methods

### Cross-sectional survey of bereaved relatives’ views

Participating organisations compiled a consecutive sample of the patients’ next-of-kin (NOK) according to eligibility criteria (Table 1) [28]. Organisations posted CODE™ information packs to the NOK three months following the patient’s death. The information pack included a:

1. covering letter
2. CODE™ questionnaire with unique identifier
3. freepost envelope for returning the questionnaire
4. information about accessing the web-based tool if on-line completion preferred.

**Table 1:** Eligibility criteria for Regional CODE™ survey

*Inclusion criteria*	
Next-of-kin to:	• A deceased adult patient (>/= 18 years of age) ○ who had died within the organisation (note: within the community setting, only patient deaths that occurred in the person’s usual place of residence were included). ○ Whose death occurred between 1st September and 30th November 2014.
*Exclusion criteria*	
	• Potential participant currently involved in a formal complaint process (to minimise additional distress). • Unexpected deaths’ were excluded (e.g. death due to an accident or suicide) in line with the National End of Life Intelligence Network approach (28) and the methodology used in the 2015 National Audit (12) • Within the hospital setting, to ensure the death was ‘expected’, the following were excluded: • Deaths </= 24 h of admission • Deaths in the Accident &Emergency department • Case of death from the following ICD-10 codes: acute myocardial infarction (I21, I22); pulmonary embolism (I26); pulmonary aneurysm (I281); sudden cardiac death (I461); aortic aneurysm (I71); injury, poisoning or external causes (S00-T98).

To remain ethically sensitive and, as this was the first time a survey of this nature had been conducted regionally, no reminder letters were sent.

### Evaluation interviews with named organisational representatives

A purposive sample of ‘named organisational representatives’ were approached to represent service leads from all three care settings (hospice, hospital and community). A letter of invitation and a Participant Information Sheet was posted to potential participants. Following written informed consent, those willing to participate, undertook a one-to-one semi-structured interview conducted by one of three experienced researchers (MG, HM, TM). Using an evaluation interview, where a ‘narrative’, conversational approach [29, 30] was employed, rather than a researcher developed evaluation form with a set of pre-determined questions and response options, was believed to promote a more participant led assessment. At the same time, it offered the opportunity for clarification and elaboration of important elements of feedback to aid understanding. These conversations were largely focused into: the current process for gaining and dealing with patient and relative feedback and complaints; reasons for participating in the project; general perspectives on taking part in the project (positives and negatives). Interviews were conducted prior to publication of the overall final report. However, an automated report of the CODE™ survey results for individual organisations was available to download via the data entry tool. This provided an opportunity for all participants to review the results for their individual organisation(s) and begin the process of action planning ahead of the final report.

An interview ‘topic guide’ was used to encourage the conversational ‘flow’ if this was not naturally occurring. Some specific areas covered were:

- Role of the interviewees
- Existing bereaved relative feedback processes within organisation (e.g. including the management and feedback of these user-views)
- Perceived levers and barriers to project participation (e.g. team format, operational processes, anticipated barriers and overcoming these).

Depending on preferences, interviews were conducted either face-to-face or via the telephone.

## Analysis

### Bereaved relatives’ survey

CODE ™ data was analysed using descriptive statistics (number, percentage) for each individual question. Median (M) and Inter Quartile Ranges (IQR) were used where appropriate. All missing data is presented within the tables (but excluded from the descriptive analysis). In terms of project feedback, each participating organisation was given their own report detailing results from their bereaved relatives’ survey. Subsequently, they were also given a report detailing how their own results compared with other organisations within the region. Within this manuscript, we provide an example of how individual organisations’ results for the key outcomes could be compared with regional and national results.

### Evaluation interviews

Each interview was audio-recorded, transcribed and analysed with a thematic approach using the “substance of the interview” [31] to formulate overarching themes and categories. One of the researchers (MG) read, and re-read the data from all 9 interviews, recorded initial impressions and developed thematic codes. Further discussion of codes was conducted with the wider team (TM, CM) to reach overall agreement on substantive categories representing the data as a whole. For the purposes of this manuscript, the codes relating to levers and barriers to project participation form the main focus.

## Results

### Response rate

From 30 eligible organisations, 19 initially agreed to participate (7 hospitals; 7 hospices; 5 community settings) although one community organisation subsequently was not able to participate due to resource issues. Of those who declined, the main reason was because they were already undertaking a bereaved relatives survey (*n* = 8); other reasons being ‘too few deaths’ within the inclusion period (*n* = 2) and one organisation simply reported that they were unable to participate on this occasion.

**Fig. 1:**
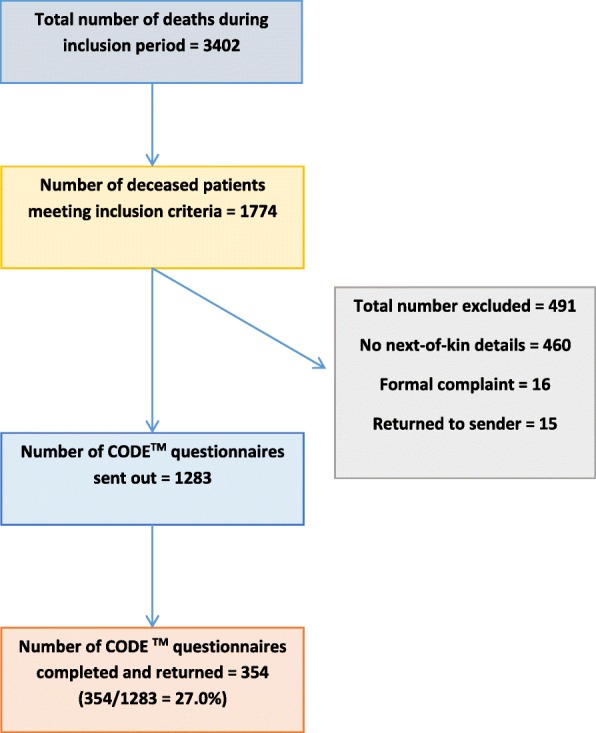
Flow chart – Response rate within the Regional CODE™ survey

From 3402 deaths, just over half (52%, *n* = 1774) met the initial inclusion criteria. Approximately a quarter of these (*n* = 491, 28%) could not subsequently be included, with the main reason due to inaccurate NOK data (Fig. 1). From 1283 CODE™ questionnaire packs sent out, 354 returned completed questionnaires (28% response rate). The hospice setting had the largest response rate (82/225, 36%) compared with other settings (hospital 218/849, 26%; community 54/209, 26%).

### Demographics (Table 2)

Overall, deceased patients were evenly split in terms of gender (*n* = 170 males, 52%) and, except for the hospice, tended to be from an older age group (*n* = 259, 70 years or older, 77%). More hospice (*n* = 74, 90%) and community (*n* = 44, 81%) patients had a cancer diagnosis compared with the hospital (*n* = 70, 32%).

**Table 2:** Demographic data for deceased patients and bereaved relatives within the Regional CODE™ survey^a^

	Hospice (*n* = 82)	Hospital (*n* = 218)	Community (*n* = 54)	All organisations (*n* = 354)
Deceased patient				
Age				
18–39	0 (0.0)	2 (1.0)	0 (0.0)	2 (0.6)
40–59	18 (22.8)	13 (6.2)	5 (10.0)	36 (10.7)
60–69	19 (24.1)	19 (9.2)	1 (2.0)	39 (11.6)
70–79	24 (30.4)	54 (26.1)	27 (54.0)	105 (31.1)
80+	18 (22.8)	119 (57.5)	17 (34.0)	154 (45.8)
Missing	3	11	4	18
Female	37 (48.7)	101 (49.5)	19 (40.4)	157 (48.0)
Missing	6	14	4	27
Ethnicity				
White British	77 (97.5)	195 (95.6)	49 (100.0)	321 (96.7)
Other e.g. White Irish, Asian Other, Mixed White/Black	2 (2.5)	9 (4.4)	0 (0.0)	11 (3.3)
Missing	3	14	5	22
Religious affiliation				
Christian	64 (81.0)	176 (85.9)	42 (84.0)	282 (84.4)
Other e.g. Buddhist, Any other religion	1 (1.3)	5 (2.5)	1 (2.0)	7 (2.1)
None	14 (17.7)	24 (11.7)	7 (14.0)	45 (13.5)
Missing	3	13	5	20
Diagnosis – proportion cancer	74 (90.2)	70 (32.1)	44 (81.5)	188 (53.1)
Bereaved relative				
Age				
18–39	5 (6.4)	6 (2.9)	0 (0.0)	11 (3.3)
40–59	28 (35.4)	61 (29.8)	12 (24.5)	101 (30.3)
60–69	22 (27.8)	9 (28.8)	14 (28.6)	95 (28.5)
70–79	17 (21.5)	46 (22.4)	15 (30.6)	78 (23.4)
80+	7 (8.9)	33 (16.1)	8 (16.3)	48 (14.4)
Missing	3	13	5	21
Female	48 (60.8)	140 (68.0)	37 (74.0)	225 (67.2)
Missing	3	13	5	21
Relationship to patient				
Husband / wife / partner	45 (57.7)	79 (38.5)	36 (72.0)	160 (48.8)
Son / daughter	19 (24.4)	87 (42.2)	12 (24.0)	118 (35.4)
Other named category e.g. brother/ sister, parent, friend	10 (12.9)	32 (15.6)	2 (4.0)	44 (13.2)
Other	4 (5.1)	7 (3.4)	0 (0.0)	11 (3.3)
Missing	4	13	4	21
Ethnicity				
White British	77 (97.5)	201 (98.0)	47 (95.9)	325 (97.6)
Other e.g. White Irish, Asian Other, Mixed White/Black,	2 (2.5)	4 (2.0)	2 (4.1)	8 (2.4)
Missing	3	13	5	21
Religious affiliation				
Christian	62 (78.5)	171 (83.4)	47 (94.0)	280 (83.8)
Other e.g. Buddhist, Any other religion	1 (1.3)	11 (5.4)	1 (2.0)	13 (3.9)
None	16 (20.3)	23 (11.2)	2 (4.0)	41 (12.3)
Missing	3	13	4	20

^a^missing data has been presented as numbers but not included in the percentage calculations

Participating bereaved relatives tended to be female (*n* = 225, 67%) and aged between 40 and 69 years (*n* = 196, 59%). Participants tended to be the spouse or partner to the patient (*n* = 160, 59%), with the exception of the hospital setting, where participants tended to be the ‘son / daughter’ (*n* = 87, 42%) completing the questionnaire. The majority of relatives and patients in this sample were ‘White British’ and of a ‘Christian’ religious affiliation.

### CODE™ questionnaire results

#### Key outcomes (Table 3)

Overall, about three-quarters of respondents reported the patient was ‘always’ treated with dignity and respect by both doctors and nurses (doctors: *n* = 245, 72%; nurses: *n* = 257, 75%). A notable minority, primarily from the hospital setting, reported the patient was ‘never’ treated with dignity and respect by doctors (*n* = 12, 6%) and nurses (*n* = 9, 4%). The majority of bereaved relatives (*n* = 272, 82%) reported they were adequately supported in the last days of life.

**Table 3:** Key outcomes as perceived by bereaved relatives within the Regional CODE™ survey^a^

	Hospice (*n* = 82)	Hospital (*n* = 218)	Community (*n* = 54)	All organisations (*n* = 354)
*How much of the time was s/he treated with respect and dignity in the last two days of life? – by doctors?*				
Always	69 (92.0)	130 (61.0)	46 (88.5)	245 (72.1)
Most of the time	4 (5.3)	34 (16.0)	2 (3.8)	40 (11.8)
Some of the time	0 (0.0)	16 (7.5)	1 (1.9)	17 (5.0)
Never	0 (0.0)	12 (5.6)	1 (1.9)	13 (3.8)
Don’t know	2 (2.7)	21 (9.9)	2 (3.8)	25 (7.4)
Missing	7	5	3	14
*How much of the time was s/he treated with respect and dignity in the last two days of life? – by nurses?*				
Always	70 (93.3)	139 (65.0)	48 (92.3)	257 (75.4)
Most of the time	4 (5.3)	39 (18.2)	2 (3.8)	45 (13.2)
Some of the time	1 (1.3)	21 (9.8)	1 (1.9)	23 (6.7)
Never	0 (0.0)	9 (4.2)	1 (1.9)	10 (2.9)
Don’t know	0 (0.0)	6 (2.8)	0 (0.0)	6 (1.8)
Missing	7	4	2	13
*Overall, in your opinion, were you adequately supported during his/her last two days of life?*				
Yes	79 (96.3)	151 (73.7)	48 (92.3)	278 (82.0)
No	3 (3.7)	54 (26.3)	4 (7.7)	61 (18.0)
Missing	0	13	2	15
*How likely are you to recommend our Organisation to friends and family?*				
Extremely likely	71 (87.7)	78 (37.5)	34 (68.0)	183 (53.8)
Likely	9 (11.1)	54 (26.0)	14 (28.0)	77 (22.6)
Neither likely nor unlikely	0 (0.0)	34 (16.3)	1 (2.0)	35 (10.3)
Unlikely	1 (1.2)	10 (4.8)	0 (0.0)	11 (3.2)
Extremely unlikely	0 (0.0)	18 (8.7)	0 (0.0)	18 (5.3)
Don’t know	0 (0.0)	14 (6.7)	1 (1.9)	16 (4.7)
Missing	0	10	4	14

^a^missing data has been presented as numbers but not included in the percentage calculations

#### Symptom control (Table 4)

The most commonly reported symptom was ‘restlessness’ with 225 respondents (65%) perceiving their family member appeared restless ‘some’ or ‘all of the time’. A small but notable minority perceived their family member had the following symptoms ‘all of the time’ in the last days of life (‘pain’ *n* = 37, 11%; ‘restless’ *n* = 51, 15%; ‘noisy rattle’ *n* = 57, 17%). Pain and restlessness being present ‘all of the time’ was most commonly reported by hospital respondents and ‘noisy rattle’ by hospice respondents.

**Table 4:** Symptom control and communication as reported by bereaved relatives within the Regional CODE™ survey^a^

	Hospice (*n* = 82)	Hospital (*n* = 218)	Community (*n* = 54)	All organisations (*n* = 354)
*In your opinion, during the last two days, did s/he appear to be in pain?*				
Yes, all of the time	6 (7.6)	26 (12.1)	5 (9.4)	37 (10.7)
Yes, some of the time	37 (46.8)	81 (37.9)	25 (47.2)	143 (41.3)
No, s/he did not appear to be in pain	36 (45.6)	107 (50.0)	23 (43.3)	166 (48.0)
Missing	3	4	1	8
*In your view, did the doctors and nurses do enough to help relieve the pain?* ^*b*^				
Yes, all of the time	64 (84.2)	116 (64.1)	34 (73.9)	214 (70.6)
Yes, some of the time	11 (14.5)	50 (27.6)	11 (23.9)	72 (23.7)
No, not at all	1 (1.3)	15 (8.2)	1 (2.2)	17 (5.6)
Missing	3	3	1	7
N/A, s/he was not in pain	3 (3.8)	34 (15.8)	7 (13.2)	44 (12.7)
*In your opinion, during the last two days, did s/he appear to be restless?*				
Yes, all of the time	6 (7.5)	38 (17.8)	7 (13.5)	51 (14.7)
Yes, some of the time	41 (51.2)	103 (48.1)	30 (57.7)	174 (50.3)
No, s/he did not appear to be restless	33 (41.3)	73 (34.1)	15 (28.8)	121 (35.0)
Missing	2	4	2	8
*In your view, did the doctors and nurses do enough to help relieve the restlessness?* ^*b*^				
Yes, all of the time	39 (71.0)	73 (44.5)	27 (64.2)	139 (53.3)
Yes, some of the time	16 (29.0)	66 (40.2)	14 (33.3)	96 (36.8)
No, not at all	0 (0.0)	25 (15.2)	1 (2.4)	26 (10.0)
Missing	3	5	1	9
N/A, s/he was not restless	24 (30.4)	49 (23.0)	11 (20.8)	84 (24.3)
*In your opinion, during the last two days, did s/he appear to have a ‘noisy rattle’ to his/her breathing?*				
Yes, all of the time	14 (18.7)	37 (17.5)	6 (11.1)	57 (16.8)
Yes, some of the time	36 (48.0)	66 (31.3)	26 (49.1)	128 (37.8)
No, s/he did not have a ‘noisy rattle’ to his / her breathing	25 (33.3)	108 (51.2)	21 (39.6)	154 (45.4)
Missing	7	7	1	15
*In your view, did the doctors and nurses do enough to help relieve the ‘noisy rattle’ to his/her breathing?* ^*b*^				
Yes, all of the time	32 (64.0)	42 (38.5)	16 (53.3)	90 (47.6)
Yes, some of the time	15 (30.0)	47 (43.1)	9 (30.0)	71 (37.6)
No, not at all	3 (6.0)	20 (18.3)	5 (16.7)	28 (14.8)
Missing	8	10	4	22
N/A, there was no ‘noisy rattle’ to his / her breathing	24 (32.4)	99 (47.6)	20 (40.0)	143 (43.1)
*The nurses had time to listen and discuss his/her condition with me.*				
Strongly agree	50 (63.3)	68 (31.9)	30 (58.5)	148 (43.1)
Agree	26 (32.9)	78 (36.6)	16 (31.4)	120 (35.0)
Neither agree nor disagree	1 (1.3)	24 (11.3)	3 (5.9)	28 (8.2)
Disagree	2 (2.5)	27 (12.7)	2 (3.9)	31 (9.0)
Strongly disagree	0 (0.0)	16 (7.5)	0 (0.0)	16 (4.7)
Missing	3	5	3	11
*The doctors had time to listen and discuss his/her condition with me.*				
Strongly agree	55 (69.6)	63 (30.0)	31 (62.0)	149 (44.0)
Agree	24 (30.4)	87 (41.1)	14 (28.0)	125 (36.9)
Neither agree nor disagree	0 (0.0)	23 (11.0)	3 (6.0)	26 (7.7)
Disagree	0 (0.0)	21 (10.0)	1 (2.0)	22 (6.5)
Strongly disagree	0 (0.0)	16 (7.6)	1 (2.0)	17 (5.0)
Missing	3	8	4	15
*Did any of the healthcare team discuss with you whether giving fluids through a ‘drip’ would be appropriate in the last two days of life?*				
Yes	23 (29.1)	77 (36.7)	10 (18.9)	110 (32.2)
No	41 (51.9)	107 (50.0)	38 (71.7)	186 (54.4)
Don’t know	15 (19.0)	26 (12.4)	5 (9.4)	46 (13.5)
Missing	3	8	1	12
*Would a discussion about the appropriateness of giving fluids through a ‘drip’ in the last two days of life have been helpful?* ^*4*^				
Yes	27 (47.4)	83 (57.2)	14 (32.6)	124 (50.6)
No	30 (52.6)	62 (42.8)	29 (67.4)	121 (49.4)
Missing	6	19	1	26
N/A, we had these types of discussions	19 (25.0)	54 (27.1)	10 (18.9)	83 (25.3)
*Did a member of the healthcare team talk to you about what to expect when s/he was dying (*e.g. *symptoms that may arise)?*				
Yes	47 (58.8)	100 (47.2)	29 (55.8)	176 (51.2)
No	33 (41.2)	112 (52.8)	23 (44.2)	168 (48.8)
Missing	2	6	2	10
*Would a discussion about what to expect when s/he was dying have been helpful?* ^*b*^				
Yes	29 (72.5)	92 (77.3)	23 (82.1)	144 (77.0)
No	11 (27.5)	27 (22.7)	5 (17.9)	43 (23.0)
Missing	8	17	1	26
N/A, we had these types of discussions	34 (45.9)	82 (40.8)	25 (47.2)	141 (43.0)

^a^missing data has been presented as numbers but not included in the percentage calculations

^b^In addition to the ‘missing’ participants who did not provide an answer to these questions, the response options *‘N/A, was not in pain / s/he was not restless /there was no ‘noisy rattle’ to his / her breathing’* were also removed from sample when calculating the overall percentages

For those reporting that their family member had experienced pain, almost three quarters responded that enough had been done by the healthcare team to control this symptom (*n* = 214, 71%). This proportion reduced, however, to around half for restlessness and noisy rattle (*n* = 139, 53% and *n* = 90, 48% respectively). For all three symptoms, respondents perceived that symptom control was best optimised by the hospice healthcare team and least likely by the hospital healthcare team.

#### Communication (Table 4)

Overall, the majority of respondents perceived that nurses (*n* = 268, 78%) and doctors (*n* = 274, 81%) had time to listen and discuss the patient’s condition with them (answered ‘agree’ or ‘strongly agree’). Around one-fifth of hospital respondents either disagreed or strongly disagreed to the statement about nurses having time to listen (*n* = 43, 20%) and a similar proportion about doctors (*n* = 37, 18%).

In terms of detecting areas of unmet need, discussions about the appropriateness of giving clinically assisted hydration (CAH) were not routinely undertaken and occurred least frequently within the community setting. Overall, for those who hadn’t had a discussion about CAH (*n* = 245), 124 (51%) would have found these types of discussion helpful.

Around half of all respondents (*n* = 176, 51%) were told what to expect when their family member was dying. Of those who weren’t told (*n* = 187), just over three quarters (*n* = 144, 77%) perceived that these types of conversation would have been helpful.

#### Cross-comparison analysis of results between organisations

**Fig. 2:**
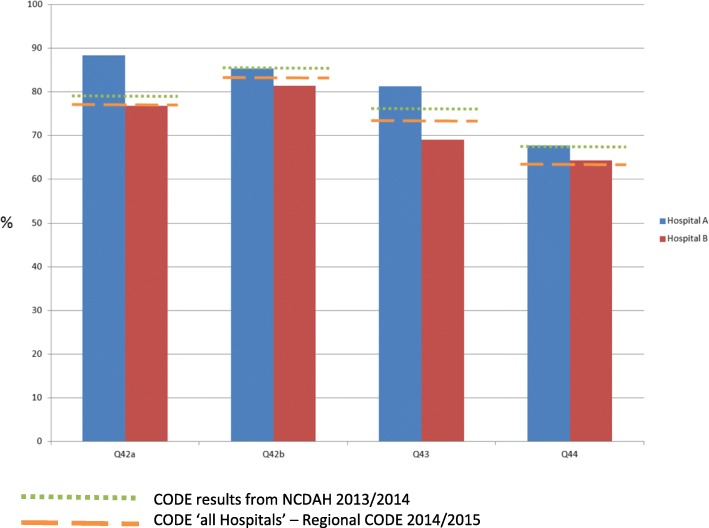
Comparison of hospital results (within one CCG) with National Care of the Dying Audit Hospital (NCDAH) and overall Regional CODETM results

Each Clinical Commissioning Group (CCG) (the organisation responsible for commissioning health services for a particular area within the region) was provided with a report of the results for its responsible provider organisations. An example of the CODE™ results for the key outcome questions for two hospitals (within a single CCG) is shown in Fig. 2.

Responses were also compared with CODE™ results from the National Care of the Dying Audit – Hospitals (NCDAH) 2013/2014 and with collated results from all participating hospitals within this Regional CODE™ survey. Comparisons suggest that perceptions about quality of care within Hospital ‘A’ tended to be higher compared with Hospital ‘B’, with NCDAH data and with collated regional results. Each organisation was then tasked with using the CODE™ results to develop action plans to help further develop clinical services.

#### Views of representatives from participating organisations

Nine individual interviews were conducted representing views across 11 services (4 hospices, 4 hospitals and 2 community settings) as one representative came from an integrated service (combined hospice / hospital /community). The interviewees’ roles were: Consultant in Palliative Medicine (*n* = 2); Clinical Nurse Specialist & Team Leader (n = 2); Clinical Services Manager (*n* = 2); Outreach Services Manager (*n* = 1), and Senior Manager (for End-of-life care / evaluation and quality) (*n* = 2). Two interviews were conducted face to face with the remainder by telephone. Interviews lasted between 35 and 60 min.

The main themes identified as ‘levers’ to present and future project participation were: ‘lack of existing feedback processes’ to obtain user-representation views about the service; ‘clear operational processes’, including a user-friendly electronic tool, written guidance and a telephone helpline; ‘significant user-feedback and opportunity to use results in a meaningful way’ which would help inform future care provision. The main theme relating to ‘barriers’ was the ‘fear of causing distress to bereaved relatives’ (which was subsequently not found to be the case in this project). Additional themes were: current ‘organisational systems not set up to capture information’; lack of organisational ‘buy in’ and lack of administrative support (Table 5).

**Table 5:** Summary of interviewees feedback about ‘Regional CODE’ – levers and barriers for participation

Levers for participation	Description	Illustrative quotes
Lack of existing feedback processes	Interviewees reported that their main reason for participation was due to a lack of existing ‘formal’ mechanisms in their own organisation for systematically gaining the views of bereaved relatives.	*‘At the moment we don’t (*referring to formal feedback processes*) …and obviously that’s another thing we’ve been looking at and our family support worker is currently looking at that.’ (P008 – Outreach Services Manager)* *‘it’s not a formal thing, it’s just, like you say, an* ad hoc *thing’ (P004 – Clinical Nurse Specialist)* ‘(having the) *opportunity to gain some feedback around our organisation really and to compare it…it* (is) *quite useful to have an understanding of where we sit with our peers’ (P008 – Outreach Services Manager)*
Clear operational processes	Interviewees fed back that the ease of participation -in terms of clear guidance and instructions to enable processes of data collection and entry - encouraged participation.	*‘the web tool itself was quite explicit. As I say, once we read it (*the guidance) *and got to understand the format of it, it seemed to run very smoothly and it was very useful and it gave us quite explicit ways of doing things....’ (P008 – Outreach Services Manager)* *‘I don’t think it could [be improved] - like I say, it was one of the better ones I’ve used, it was really easy to use’ (P004 – Clinical Nurse Specialist)* *‘it’s good to have good access to assistance, you know if I ring you or email you, you reply, and to know that makes it possible to do it...otherwise I wouldn’t have completed it’ (P002 – Consultant in Palliative Medicine)*
Significant user-feedback and opportunity to use results in meaningful way	Interviewees perceived that data generated would provide valuable local information to better understand experiences of care in the last days of life and provide a direction on which to base the improvement of future services and care delivery.	*‘we will…..create reports that go to the senior management team and it’ll go to the trustees, so that we’re looking at… what are we’re doing well at and….things we can improve on and what (we are) going to do.’ (P001 – Clinical Services Manager)* *‘I think this is extremely good leverage to make people sit up and understand the changes that are required really and provide evidence for regulators but (also) our patients’ (P009 – Consultant in Palliative Medicine)* *‘It will be taken to our governance group and then it will be fed out more widely to the rest of the teams.’ (P008 – Outreach Services Manager)*
Barriers for participation	Description	Illustrative quotes
Fear of causing distress to bereaved relatives	The main concern reported by interviewees related to the potential distress to bereaved relatives when asking them to participate in the survey.	*‘Yeah I mean obviously you also have worries of barriers that you kind of fear of being maybe a bit intrusive and I think sometimes you worry that you rekindle maybe thoughts and feelings in bereaved relatives after a period of time and I think those are kind of our personal worries as members of staff I think’* (*P008 – Outreach Services Manager*) *‘No...complaints came back...no letters...no telephones...there was nothing...we didn’t seem to have any that, you know...maybe people had opened and thought “Oh, I don’t know” - they all came within a short space of time. It was actually really quite good to see that people received it, thought about it, wanted to do it, and sent them back. And I thought that the number that we got returned was actually a high rate of return’ (P001 – Clinical Services Manager)*
Organisational systems not set up to capture information	Some interviewees reported that they were hindered by their organisational information technology systems and processes, e.g. multiple systems; information not being routinely available; accuracy of information not assured requiring additional work to confirm details.	*‘I addressed all the envelopes myself just so I knew that it had gone to the right people and that I couldn’t blame anybody else if it went wrong’ (P004 – Clinical Nurse Specialist)*
Lack of organisational ‘buy in’	Some interviewees reported strong ‘buy in’ from senior and executive management ensured the project was seen as a priority. Participation was potentially compromised if lacking in senior support.	*(*successful participation in Quality Assurance Project relied on*)’...the good will of staff who are involved...because it’s not actually part of anyone’s particular role if you know what I mean...I think that it is because of our [non managerial staff] drive to try and ascertain the views of the families, but that hasn’t come from the board it’s come from within our levels to say how can we evidence what we do’ (P005 – Clinical Nurse Specialist)* *‘Once I had engagement of the deputy medical director, I knew it [project participation] would happen’ (P009 – Consultant in Palliative Medicine)*
Lack of administrative support	Interviewees who cited having a small ‘team’ designated to support the project described a more positive experience, and without administrative support participation would have been compromised.	*‘I was over-worrying about the time it would all take and the inputting onto the system, more so for the admin staff...they were really, really good and they accepted that I was explaining that this was important to us to be involved’ (P001 – Clinical Services Manager)* *‘If I had to do it again...it wouldn’t just be me. I would be much more autonomous about this is...I need other people, you know, I need to bring other people with me so it can’t just be in isolation’ (P003 – Senior Manager)*

## Discussion

Overall, this was the first bereaved relatives’ survey spanning across three healthcare settings undertaken within this specific English region. The ability to engage with 18 (60%) of all organisations, and the fact that the main reason for non-participation was due to current participation in bereaved relatives’ surveys, was testament to the success of this quality assurance initiative. This model of providing quality assurance is one which could be replicated within other regions and across countries as a whole. Providing opportunities for user feedback towards services is a key health care priority both nationally and internationally. Within the UK, this commitment is outlined within the NHS constitution [32] and NHS Mandate [33]. Improving support to and engagement with bereaved relatives is also a key objective within the Next Steps On The NHS Five Year Forward View [34]. Results from this project confirm that the majority of these regional organisations did not have formal mechanisms to capture bereaved relatives’ views. CODE™ is a valid and reliable tool which could bridge this clinical need and support organisations to obtain feedback of this nature. Interviewees reported that the data generated was perceived to provide valuable local information to better understand experiences of care in the last days of life. Additionally, the opportunity to subsequently benchmark their results with others regionally was seen as a positive outcome.

Generally, the majority of bereaved relatives reported good perceptions of care, although a small but significant minority reported poor experiences. Those who experienced care within the acute hospital settings were more likely to report issues in terms of the care received or the level of family support provided. Motivations for organisations’ participation could have been influenced by the expectation that bereaved relatives’ perceptions were likely to be favourable. Responses, however, demonstrated variability in the care provided, implying that the motivation was to genuinely seek user-feedback about their service. Although concerns were raised by interviewees about potential distress to bereaved relatives, in reality there were few complaints from bereaved relatives who participated in this project. This finding would reinforce previous research suggesting that bereaved relatives are keen to be given the opportunity to engage in research due to a number of different motivations [35, 36].

The project was not without limitations. There was a high proportion of missing or inaccurate NOK details and, as it was imperative the CODE™ questionnaire was sent to the most appropriate individual, this limited the total number of potential participants. Additionally, this may have led to selection bias as, within some organisations, those who were engaged with Specialist Palliative Care services were more likely to have their details documented accurately. The wider implications of inaccurate details include the potential impact on providing bereavement support if NOK can’t be accurately contacted. By being involved with the quality assurance project, however, this helped highlight to individual organisations some of the limitations of their internal reporting mechanisms and facilitated the potential development of solutions in these organisations.

Overall the response rate of returned and completed CODE ™ questionnaires (27%) was lower than expected (estimated to be 35–40%). It was also lower compared with previous studies of this nature including those which have used CODE™ [14, 18]. One reason for the lower response rate was the decision at the project outset not to use reminder letters, a method recognised to increase response rates [23]. Ongoing developments for the CODE™ questionnaire include further Public and Patient engagement to ascertain the best methods to approach and recruit potential participants for these types of surveys [19].

We did not obtain the demographic details of non-responders. This would have enabled a comparison between respondents and non-responders and provided information about the representativeness of our sample compared with the population as a whole. This is especially pertinent due to the fact that most participants were of white ethnic origin and stated they had a ‘Christian’ religious affiliation. The key element of the project, however, was to assess whether it was possible to engage with clinical services and hence a balance needed to be met between making it sufficiently feasible for individual organisations to participate versus the ideal methodology for conducting a survey of this nature.

Overall, perceptions about hospital care were poorer compared with hospice and community settings especially in terms of symptom control and communication. These areas are recognised themes from patients and family carer feedback [37] although for pain control, findings from previous studies have varied. One recent study demonstrated that those who died in hospital were more likely to have experienced pain compared with those who died at home [38] but others have not supported this finding [39]. It is noteworthy, however, that the deceased patient populations differed between care settings, with hospitals having a greater prevalence of non-cancer patients. In the most recent UK National VOICES survey, findings also suggested that pain control was potentially better met for those with a cancer diagnosis compared with those dying from non-malignant disease [5].

The greatest proportion of discussions about fluids at the end-of-life was reported by the hospital participants and the lowest within the community setting. This may relate to the fact that it is more challenging to provide clinically assisted hydration (CAH) in home or care home facilities. And in turn, healthcare professionals may not have broached this issue due to these types of concerns. The greatest degree of unmet information need about CAH was in the hospital setting, although across all settings, participants would have appreciated discussions. This may be in part due to the generally positive views of CAH by family members [40, 41] and the symbolic meaning that it brings [42]. It is important to understand that making ‘blanket’ assumptions about the appropriateness of engaging in such discussions cannot be supported, and sensitive communication, using open screening questions is likely to be required to promote the provision of individualised and responsive care [43].

CODE™ specifically focuses on care of patients in the last days of life and family support, and this is the first time it has been used to assess the quality of care in a number of different healthcare settings (hospice, hospital and community). Due to the methods of questionnaire administration, the survey allows each organisation to have individual and personalised feedback about bereaved relatives’ perceptions of care. Previous national surveys of this nature have tended to feedback at a regional or CCG area level [5]. Additionally, the project subsequently enabled individual organisations to ‘benchmark’ themselves with findings from other similar organisations which offers the opportunity to facilitate cross-site learning. Although not conducted within the scope of this project, future follow-up interviews with the representatives from participating organisations would be beneficial to establish the key actions undertaken to help improve their clinical services. Additionally, future developments may include the opportunity to repeat the post-bereavement survey to assess the impact of changes over time. By assessing the feasibility that an evaluation such as this was possible within this region, a future post-bereavement evaluation would offer the opportunity to undertake more robust survey methodology to help further address any confounding factors.

Some of the real strengths of this project include the ability for individual organisations to:

- demonstrate active engagement with bereaved relatives and have user-views about their service; this is particularly pertinent to provide evidence for the regulator organisations such as the ‘Care Quality Commission’ within the UK
- have a quick ‘at a glance’ report to highlight the strengths of the organisation and areas that require further development
- compare their own findings with other similar organisations as a whole and with others within their locality; additionally, for hospitals being able to ‘benchmark’ their data with previous national findings
- utilise the CODE™ findings to create action plans to enact service improvement initiatives as well as facilitate learning from other organisations [26, 27].

## Conclusions

With the national recommendations to use individualised care plans for dying patients, the optimal way to evaluate the impact of these and provide assurance that care is being delivered in a timely and sensitive manner should be determined. Post-bereavement questionnaires are recognised to be a useful way to do this and CODE™ represents a valid and reliable outcome measure to use. The successful engagement with the project is testament to the commitment of staff and organisations with the clear desire to seek user-feedback to improve care. The model of evaluation and the use of a ‘benchmarking’ approach, could be used at a local, national and international context to help drive an ongoing continuous quality improvement programme to improve care for dying patients.

## Abbreviations

CAH: Clinically Assisted Hydration
CCG: Clinical Commissioning Group
CODE: Care Of the Dying Evaluation
ECHO-D: Evaluating Care and Health Outcomes – for the Dying
IQR: Inter Quartile Ranges
LCP: Liverpool Care of the Dying Pathway
M: Median (M)
NCDAH: National Care of the Dying Audit – Hospitals
NOK: next-of-kin
